# The Proteome Response to Amyloid Protein Expression *In Vivo*

**DOI:** 10.1371/journal.pone.0050123

**Published:** 2012-11-21

**Authors:** Ricardo A. Gomes, Catarina Franco, Gonçalo Da Costa, Sébastien Planchon, Jenny Renaut, Raquel M. Ribeiro, Francisco Pinto, Marta Sousa Silva, Ana Varela Coelho, Ana Ponces Freire, Carlos Cordeiro

**Affiliations:** 1 Instituto de Tecnologia Química e Biológica, Universidade Nova de Lisboa, Oeiras, Portugal; 2 Centro de Química e Bioquímica, Faculdade de Ciências da Universidade de Lisboa, Lisboa, Portugal; 3 Département of Environnent and Agrobiotechnologies, Centre de Recherche Public Gabriel Lippmann, Belvaux, Luxembourg; University of Kent, United Kingdom

## Abstract

Protein misfolding disorders such as Alzheimer, Parkinson and transthyretin amyloidosis are characterized by the formation of protein amyloid deposits. Although the nature and location of the aggregated proteins varies between different diseases, they all share similar molecular pathways of protein unfolding, aggregation and amyloid deposition. Most effects of these proteins are likely to occur at the proteome level, a virtually unexplored reality. To investigate the effects of an amyloid protein expression on the cellular proteome, we created a yeast expression system using human transthyretin (TTR) as a model amyloidogenic protein. We used *Saccharomyces cerevisiae*, a living test tube, to express native TTR (non-amyloidogenic) and the amyloidogenic TTR variant L55P, the later forming aggregates when expressed in yeast. Differential proteome changes were quantitatively analyzed by 2D-differential in gel electrophoresis (2D-DIGE). We show that the expression of the amyloidogenic TTR-L55P causes a metabolic shift towards energy production, increased superoxide dismutase expression as well as of several molecular chaperones involved in protein refolding. Among these chaperones, members of the HSP70 family and the peptidyl-prolyl-cis-trans isomerase (PPIase) were identified. The latter is highly relevant considering that it was previously found to be a TTR interacting partner in the plasma of ATTR patients but not in healthy or asymptomatic subjects. The small ubiquitin-like modifier (SUMO) expression is also increased. Our findings suggest that refolding and degradation pathways are activated, causing an increased demand of energetic resources, thus the metabolic shift. Additionally, oxidative stress appears to be a consequence of the amyloidogenic process, posing an enhanced threat to cell survival.

## Introduction

Protein misfolding and aggregation are common features in many neurodegenerative amyloid disorders, such as Alzheimer, Parkinson and transthyretin amyloidosis (ATTR) [1], [2]. In each case, a specific amyloidogenic protein misfold and follows a toxic aggregation pathway leading to a defined clinical outcome [1], [2]. A legion of factors that may trigger protein misfolding and aggregation has been implied in the development of this kind of pathologies, such as abnormal proteolysis, point mutations and post-translational modifications, namely phosphorylation, oxidation and glycation [2], [3]. Cell quality control mechanisms evolved to cope with protein misfolding and aggregation, including molecular chaperones and protein degradation pathways that prevent protein aggregation by either protein refolding or degradation [4]–[6]. However, in the disease process these mechanisms are not sufficient to prevent the accumulation of toxic protein aggregates and recent studies have implicated several components of the protein quality control system in neurodegenerative disorders of amyloid type (reviewed in [5], [6]). Thus, a decreased cell capacity to clear misfolded proteins may be directly involved in pathogenesis. In fact, one hypothesis to explain the late onset of several conformational disorders considers the loss of effectiveness of the protein quality control system with age, either arising from environmental insults, mutations or unidentified triggers [7]. Although the regulation of these pathways has been considered a therapeutic strategy [8] our knowledge of these processes is still very limited and therefore a deeper understanding of the cellular response mechanisms, at the proteome level, to misfolding and aggregation of amyloidogenic proteins is of great interest. Importantly, even in familial neuropathies, where genetic determinants play a key role, several observation points to the involvement of non-genetic factors. In ATTR, where the amyloid deposits are mainly composed of transthyretin (TTR), several TTR amyloidogenic point mutations with different degrees of amiloidogenicity (i.e., different tendency to misfold and aggregate) are associated to disease onset and progression [9], [10]. However, it was observed that many mutation carriers are asymptomatic throughout their lives and non-mutant TTR also forms amyloid, causing senile systemic amyloidosis [11]–[13]. Additionally, patients carrying the same mutation show a wide range of age at onset from 20 to 70 years [14]. We recently discovered that ATTR patients show increased protein glycation that decreases the chaperone activity of fibrinogen, a TTR protein binding partner, hence promoting transthyretin unfolding and aggregation in ATTR [15].

In this work we investigated the cellular responses to the misfolding and aggregation of an amyloidogenic protein using a high-throughput proteomics approach to screen differentially expressed proteins. For that purpose, we used yeast *Saccharomyces cerevisiae* as a host to express human TTR, used as a model amyloidogenic protein. As the general protein quality control systems are highly conserved, especially in eukaryotes, yeast is a valuable model to evaluate fundamental aspects of protein misfolding and the molecular mechanisms involved in the cellular response. Additionally, *Saccharomyces cerevisiae* was the first eukaryote to have its genome sequenced and, recently, its entire proteome has been mapped [16], [17]. Yeast has been successfully used to investigate fundamental aspects of protein misfolding involved in syndromes like amyotrophic lateral sclerosis, Huntington’s disease and Parkinson’s disease [18]–[20]. TTR was investigated as an amyloidogenic protein for two main reasons: first, it is one of the best structurally characterized amyloidogenic proteins and second, several known point mutations are associated to different degrees of amiloidogenicity and disease progression. This allows the discrimination between the cell response to the expression of a heterologous protein and the amyloidogenic version of the same protein. Thus, wild-type TTR and the highly amyloidogenic TTR variant L55P (leucine for proline at position 55) were selected for expression in yeast. Although these two variants only differ in one amino acid, TTR-L55P has a higher intrinsic propensity to misfold and aggregate and carriers of this amyloidogenic mutation have a very early age at disease onset [21].

Differential proteome changes between the control (cells carrying the empty plasmid), cells expressing TTR-wt (BTTR-wt) and cells expressing TTR-L55P (BTTR-L55P) were analyzed by 2D-DIGE coupled to protein identification by tandem MS. Expression of each TTR forms has no toxic effects on yeast and does not impair cell division, population growth and metabolism, despite the formation of TTR aggregates in yeast cells expressing the L55P variant. About 20 proteins were found to be differentially expressed in BTTR-wt while 70 were differentially expressed in BTTR-L55P. Changes in proteins involved in folding and degradation processes were detected, together with an increased expression of translation pathways. Major changes at the proteome level were associated with increased carbohydrate, energy and amino acid metabolism.

## Results and Discussion

### Protein Expression Analysis by 2D-DIGE

**Figure 1:**
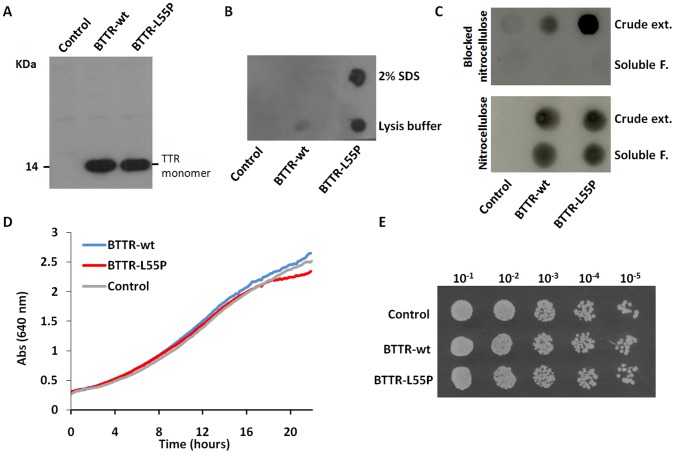
Characterization of TTR expression in yeast. (A) Relative quantitation of TTR-wt and TTR-L55P expression by Western blot with anti-human TTR antibody. Since TTR-L55P aggregates, electrophoretic separation of proteins was performed in the presence of urea to compare the total protein amount. TTR monomer is observed with molecular masses of approximately 14 kDa only in cells expressing TTR (BTTR-wt and BTTR-L55P). Similar expression levels were observed for the two TTR variants. (B) Protein aggregate filtration assay of insoluble protein fraction. TTR aggregates insoluble in 2% SDS were observed only in yeast cells expressing the amyloidogenic L55P variant (BTTR-L55P). This indicates that TTR-L55P when expressed in yeast forms high molecular mass amyloid-like aggregates, in contrast to TTR-wt. A substantially higher TTR amount was also found in the insoluble protein fraction of BTTR-L55P in comparison with BTTR-wt. (C) Control assay. The crude extract and the soluble protein fraction were analysed through a nitrocellulose membrane (as a regular dot-blot assay) and a nitrocellulose membrane that was blocked prior to the filtration procedure. Growth curves (D) and dilution spot assay (E) of yeast cells expressing the TTR variants and the control shows that, although the highly amyloidogenic TTR variant L55P was expressed in yeast, no changes were detected in yeast cell growth and cell viability.

To quantitatively investigate proteome changes of yeast cells in response to TTR expression we analysed and compared the proteomes of yeast BY4741 cells carrying the plasmid without the insert (control), TTR-wt (BTTR-wt) and TTR-L55P variant (BTTR-L55P). TTR expression was confirmed by MS analysis (data not shown) and western blot, where similar expression levels of TTR-wt and TTR-L55P were detected (Figure 1A). To analyse the presence of TTR aggregates, a protein aggregate filtration assay was performed (Figure 1B). This microfiltration method is based on the finding that high-molecular mass amyloid-like aggregates are SDS-insoluble, being therefore retained in a 0.2 µm blocked membrane. In fact, if the nitrocellulose membrane is blocked previously to the filtration procedure, TTR-containing insoluble protein aggregates are retained while soluble TTR do not bind to the blocked membrane, in contrast to a regular dot-blot assay using non-blocked nitrocellulose membrane (Figure 1C). Thus this method is indeed a protein aggregation filter trap assay. Using blocked nitrocellulose, a positive signal was only obtained in the crude extract indicating the presence of TTR in the insoluble fraction. As shown in Figure 1B, TTR aggregates insoluble in 2% SDS were clearly observed in yeast cells expressing the amyloidogenic L55P variant (BTTR-L55P). SDS-insoluble aggregates were not detected in BTTR-wt and, as expected, in the control (Figure 1B). In addition, a substantially higher TTR amount was found in the insoluble protein fraction of BTTR-L55P in comparison to BTTR-wt (Figure 1B and C). Thus, even though no cell toxicity and growth defects were observed (Figure 1D and E), TTR-L55P variant, when expressed in yeast, forms high-molecular mass amyloid-like aggregates.

**Figure 2:**
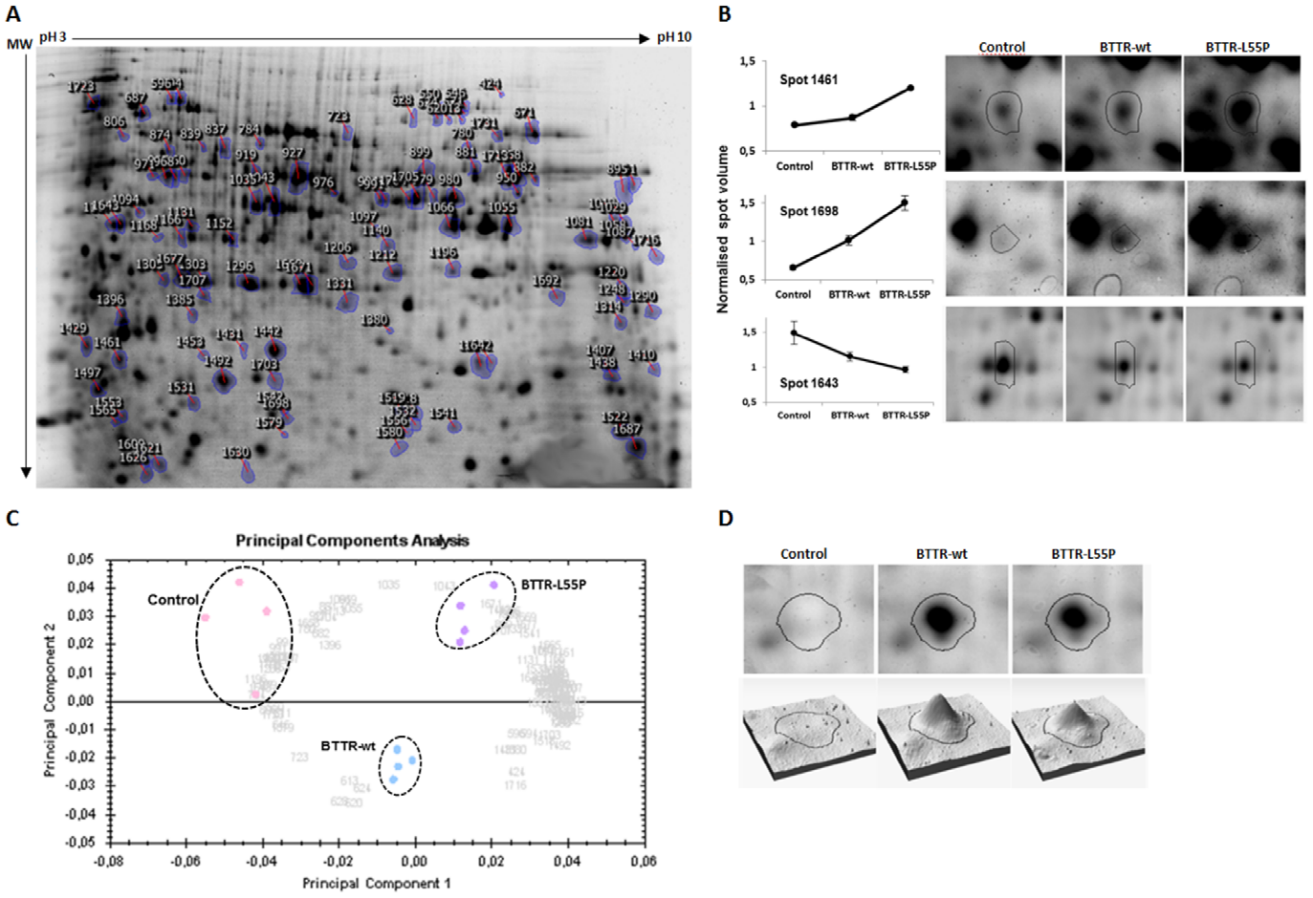
2D-DIGE differential protein expression analysis. (A) Representative 2D-DIGE gel image showing the spot map corresponding to the internal standard (Cy2 labeling), which is common to all gels analyzed. Sample preparation and labeling were performed as described in the material and methods section. Indicated spots showed a statistically significant variation of spot volume with 95% confidence level (p<0.05) and a minimal fold variation of 1.3. These spots were removed for subsequent protein identification by mass spectrometry (see Table 1 for code assignment). (B) Example of one protein present in higher abundance only in BTTR-L55P (Ubiquitin-like protein SMT3, spot 1461), one protein with an increase abundance in BTTR-wt and BTTR-L55P (FK506-binding protein, spot 1698) and one protein present in lower abundance both in BTTR-wt and BTTR-L55P (60 S acidic ribosomal protein P0, spot 1643). The spots of interested are encircled and the graphs represent the normalized spot volume. The spot volumes are an average of the 4 biological replicates used for each experimental group. (C) PCA of the 2D-DIGE results. Each data point in the PCA plot represents the global expression values for all spots with a significant ANOVA value (p<0.05). The PCA clustered the 6 individual Cy3 and Cy5-labeled 2D-gel images into three discreet groups differentiated by two principle components PC1 and PC2, explaining a cumulative 82% of all the variation. A separation between the control (carrying the empty plasmid), cells expressing the non-amyloidogenic TTR-wt (BTTR-wt) and the amyloidogenic variant TTR-L55P (BTTR-L55P) is clearly observed. Replicate samples were encircled manually for illustration. (D) 2D-DIGE image analysis of the protein spot identified as human TTR. Figure shows the spot expression map and three-dimensional spot image for each experimental group.

The 2D electrophoretic maps contained around 1800 spots (Figure 2A). From these, a total of 78 protein spots were detected with a statistically significant change in abundance (ANOVA p<0.05) with an absolute variation ≥1.3-fold from at least one experimental group. Examples of three spot patterns are shown in Figure 2B. Spot 1461 increase its abundance only in BTTR-L55P while spot 1643 decrease its abundance in both BTTR-wt and BTTR-L55P. The spot 1698 is differentially expressed in both experimental groups but shows a significantly higher abundance in BTTR-L55P compared to BTTR-wt. A principal component analysis (PCA) analysis shows that 2D gel images cluster into three well separated groups (Figure 2C), indicating a clear differentiation between the expression of the non-amyloidogenic TTR-wt and the amyloidogenic TTR-L55P form with significant changes in protein abundances. In fact, by directly comparing the 2D-DIGE maps of BTTR-wt and BTTR-L55P with the control, a total of 24 and 75 spots, respectively, were differentially expressed, highlighting a much higher induced change upon expression of the amyloidogenic TTR variant. Interestingly, of the 24 protein spots with changes in abundance between BTTR-wt/control, 21 spots were also differentially expressed in BTTR-L55P/control with a similar fold variation. These changes are likely to be due to the heterologous TTR expression and not because of TTR misfolding. However, 54 protein spots exhibited significant changes in abundance exclusively upon TTR-L55P expression, suggesting that relevant proteins involved in the cell response to TTR misfolding and aggregation are revealed with this approach.

**Table 1:** Differentially expressed proteins identified by MALDI-TOF-TOF MSMS.

Gene symbola)	Protein names	Control/BTTR-wt		Control/BTTR-L55P		Functionb)	Acces. codec)	Spotno.d)	MS datae)		
		Fold Var.	P value	Fold Var.	P value				Prot. Score	Pep. score	#Matched MSMS peptides
ACON	**Aconitatehydratase, mitochondrial**	+1.7	6.39E-03	+1.4	5.64E-03	CM	P19414	424	321	249	4
ADH1	**Alcohol dehydrogenase 1**	−1.6	7.84E-03	−1.5	1.24E-02	CM	P00330	991	617	504	9
ADH1	**Alcohol dehydrogenase 1**	−1.5	1.07E-03	−1.4	1.19E-02	CM	P00330	994	704	563	9
ADH3	**Alcohol dehydrogenase 3, mitochondrial**	+1.4	1.23E-02	+1.5	2.75E-03	CM	P07246	950	977	774	10
ADK	**Adenosine kinase**	–	–	+1.4	3.62E-03	NM	P47143	1094	537	429	4
AIM29	**Altered inheritance rate of mitochondria protein 29**	−1.4	8.26E-03	−1.4	1.29E-02	U	P36154	1453	121	76	2
ALF	**Fructose-bisphosphatealdolase**	–	–	+1.3	5.18E-03	CM	P14540	1035	221	130	3
ALF	**Fructose-bisphosphatealdolase**	–	–	+1.3	1.85E-02	CM	P14540	1131	277	168	3
ATPA	**ATP synthase subunit alpha, mitochondrial**	−1.4	1.29E-03	–	–	EM	P07251	780	647	484	6
ATPB	**ATP synthase subunit beta, mitochondrial**	–	–	+1.5	3.19E-04	EM	P00830	971	639	519	8
BCA1	**Branched-chain-amino-acid aminotransferase, mithocondrial**	–	–	+1.6	2.60E-04	AM	P38891	1565	130	110	2
BCA2	**Branched-chain-amino-acid aminotransferase, cytosolic**	–	–	+1.6	2.60E-04	AM	P47176	1565	220	199	2
BMH2	**Protein BMH2**	–	–	−1.4	8.98E-04	PFD	P34730	1127	505	395	6
CISY1	**Citrate synthase, mitochondrial**	−1.4	1.11E-02	+1.5	2.31E-01	CM	P00890	881	167	118	2
COX14	**Cytochrome c oxidase assembly protein COX14**	–	–	+1.5	1.62E-02	U	P39103	1532	104	77	1
CYPH	**Peptidyl-prolylcis-trans isomerase**	–	–	+1.7	7.20E-03	PFD	P14832	1519	193	133	3
DHE4	**NADP-specific glutamate dehydrogenase 1**	–	–	+1.6	3.17E-06	EM	P07262	837	977	771	9
DLD3	**D-lactate dehydrogenase [cytochrome] 3**	−1.4	1.29E-03	–	–	CRH	P39976	780	1020	797	8
DTD	**D-tyrosyl-tRNA(Tyr) deacylase**	–	–	+1.4	2.59E-02	T	Q07648	1642	84	63	2
DUG1	**Cys-Glymetallodipeptidase DUG1**	–	–	+1.6	3.17E-06	PFD	P43616	837	343	303	4
EF1A	**Elongation factor 1-alpha**	–	–	+1.4	2.82E-03	T	P02994	891	802	692	8
EF1A	**Elongation factor 1-alpha**	+1.3	1.82E-02	+1.5	2.60E-03	T	P02994	895	129	88	2
EF1A	**Elongation factor 1-alpha**	+1.4	7.81E-03	+1.6	7.65E-04	T	P02994	1013	294	238	4
EF1A	**Elongation factor 1-alpha**	–	–	+1.4	4.58E-03	T	P02994	1029	846	714	8
EF1B	**Elongation factor 1-beta**	–	–	+1.5	7.07E-05	T	P32471	1429	236	222	3
EF2	**Elongation factor 2**	–	–	+1.3	1.27E-03	T	P32324	1621	86	78	2
EIF3G	**Eukaryotic translation initiation factor 3 subunit G**	–	–	−1.3	1.85E-02	T	Q04067	1097	712	570	8
ENO1	**Enolase 1**	–	–	+1.5	3.07E-02	CM	P00924	1542	618	567	6
ENO2	**Enolase 2**	–	–	+1.5	3.07E-02	CM	P00925	1542	532	501	6
FHP	**Flavohemoprotein**	+1.6	1.75E-03	+1.8	7.61E-06	CRH	P39676	976	704	618	7
FKBP	**FK506-binding protein 1**	+1.4	1.91E-04	+2.2	4.90E-04	PFD	P20081	1698	163	110	3
G3P2	**Glyceraldehyde-3-phosphate dehydrogenase 2**	–	–	+1.6	5.88E-03	CM	P00358	1609	226	211	3
G3P3	**Glyceraldehyde-3-phosphate dehydrogenase 3**	–	–	+1.6	5.88E-03	CM	P00359	1609	226	211	3
GBLP	**Guanine nucleotide-binding protein subunit beta-like protein**	–	–	+1.5	7.49E-03	T	P38011	1541	315	223	3
GBLP	**Guanine nucleotide-binding protein subunit beta-like protein**	–	–	+1.4	8.16E-04	T	P38011	1556	108	92	1
GPP1	**(DL)-glycerol-3-phosphatase 1**	–	–	−1.3	3.89E-03	LM	P41277	1152	479	343	6
HIS2	**Histidine biosynthesis trifunctional protein**	+1.4	4.50E-03	+1,5	3.63E-04	AM	P00815	1692	419	367	6
HSP10	**10 kDa heat shock protein, mitochondrial**	+1.7	9.55E-03	+1.8	1.15E-03	PFD	P38910	1522	465	347	4
HSP60	**Heat shock protein 60, mitochondrial**	+1.6	8.31E-03	+1.8	2.12E-03	PFD	P19882	687	383	204	4
HSP60	**Heat shock protein 60, mitochondrial**	–	–	+1.6	1.86E-02	PFD	P19882	1438	369	307	4
HSP71	**Heat shock protein SSA1**	–	–	+1.4	2.62E-03	PFD	P10591	960	161	51	2
HSP72	**Heat shock protein SSA2**	+1.3	1.40E-02	+1.5	2.88E-03	PFD	P10592	874	165	127	2
HSP72	**Heat shock protein SSA2**	+1.4	8.91E-03	+1.6	4.37E-04	PFD	P10592	967	272	135	3
HSP75	**Heat shock protein SSB1**	–	–	−1.5	4.53E-03	PFD	P11484	613	177	138	4
HSP77	**Heat shock protein SSC1, mitochondrial**	+1.6	1.23E-02	+1.4	1.28E-02	PFD	P12398	594	310	228	6
HSP77	**Heat shock protein SSC1, mitochondrial**	+1.4	2.27E-02	–	–	PFD	P12398	596	329	196	6
IDHP	**Isocitrate dehydrogenase [NADP], mitochondria**	+2.5	7.38E-03	+2.1	1.18E-02	CM	P21954	1703	139	92	2
IF4A	**ATP-dependent RNA helicase eIF4A**	−2.2	2.09E-02	–	–	T	P10081	1396	170	111	3
IF4B	**Eukaryotic translation initiation factor 4B**	–	–	+1.5	9.26E-05	T	P34167	839	358	284	5
ILV5	**Ketol-acid reductoisomerase, mitochondrial**	–	–	−1.3	6.76E-03	AM	P06168	980	565	414	7
ILVB	**Acetolactate synthase catalytic subunit, mitochondrial**	–	–	−1.3	1.98E-02	AM	P07342	571	152	87	4
INO1	**Inositol-3-phosphate synthase**	–	–	+1.5	8.65E-04	CM	P11986	1168	282	255	3
KPYK1	**Pyruvate kinase 1**	–	–	+1.5	4.33E-03	CM	P00549	1140	345	197	4
MPCP	**Mitochondrial phosphate carrier protein**	+2.4	5.97E-03	+1.5	2.05E-02	TP	P23641	1716	335	286	4
NDK	**Nucleoside diphosphate kinase**	–	–	+1.3	3.39E-02	NM	P36010	1407	294	288	5
PDC1	**Pyruvate decarboxylase isozyme 1**	–	–	−1.3	1.30E-01	CM	P06169	723	1390	1222	11
PDI	**Protein disulfide-isomerase**	–	–	+1.3	1.05E-02	PFD	P17967	1723	474	391	5
PGK	**Phosphoglycerate kinase**	–	–	+1.4	2.67E-02	CM	P00560	1220	457	348	5
PGK	**Phosphoglycerate kinase**	+1.5	1.75E-02	+1.6	1.62E-03	CM	P00560	1248	511	431	6
PUR92	**Bifunctional purine biosynthesis protein ADE17**	–	–	+1.3	1.44E-02	NM	P38009	628	349	215	3
PYRF	**Orotidine 5'-phosphate decarboxylase**	−1.4	1.97E-02	−1.5	5.28E-03	NM	P03962	1212	185	75	2
PYRF	**Orotidine 5'-phosphate decarboxylase**	–	–	+1.5	2.64E-03	NM	P03962	1385	86	63	2
RL11A	**60S ribosomal protein L11-A**	–	–	+1.5	7.07E-05	T	P0C0W9	1429	565	482	6
RL11B	**60S ribosomal protein L11-B**	–	–	+1.5	7.07E-05	T	Q3E757	1429	565	482	6
RL12	**60S ribosomal protein L12**	–	–	+1.3	5.32E-03	T	P17079	1410	125	112	2
RLA0	**60S acidic ribosomal protein P0**	–	–	−1.5	2.02E-03	T	P05317	1643	443	358	6
RS12	**40S ribosomal protein S12**	–	–	−1.4	3.05E-02	T	P48589	1497	307	271	4
RS3	**40S ribosomal protein S3**	+1.7	9.55E-03	+1.8	1.15E-03	T	P05750	1522	212	199	2
RS5	**40S ribosomal protein S5**	–	–	−1.3	2.65E-05	T	P26783	1196	261	153	3
SAM2	**S-adenosylmethionine synthase 2**	–	–	+1.5	3.19E-04	AM	P19358	971	253	191	3
SMT3	**Ubiquitin-like protein SMT3**	–	–	+1.5	5.90E-06	PFD	Q12306	1461	163	146	3
SODC	**Superoxide dismutase [Cu-Zn]**	–	–	+1.4	9.64E-03	CRH	P00445	1442	363	247	4
SYDC	**Aspartyl-tRNAsynthetase, cytoplasmic**	–	–	+1.3	1.44E-02	T	P04802	628	301	228	5
SYRC	**Arginyl-tRNAsynthetase, cytoplasmic**	–	–	−1.3	5.76E-02	T	Q05506	620	245	164	3
SYRC	**Arginyl-tRNAsynthetase, cytoplasmic**	–	–	−1.4	4.33E-02	T	Q05506	624	473	333	5
TKT1	**Transketolase 1**	–	–	−1.3	1.73E-03	CM	P23254	550	322	296	5
TPIS	**Triosephosphateisomerase**	–	–	+1.4	1.61E-02	CM	P00942	1296	432	342	5
TPIS	**Triosephosphate isomerase**	–	–	+1.5	7.15E-03	CM	P00942	1669	906	750	7
TPIS	**Triosephosphate isomerase**	–	–	+1.4	6.10E-02	CM	P00942	1671	513	443	4
TTR	**Human transthyretin**	–	–	–	–	–	P02766	1492	592	534	6
UBX1	**UBX domain-containing protein 1**	–	–	−1.4	1.65E-03	PFD	P34223	806	682	562	7
VDAC1	**Mitochondrial outer membrane protein porin 1**	–	–	+1.4	2.67E-02	EM	P04840	1220	171	135	3
YBD6	**UPF0001 protein YBL036C**	–	–	−1.5	4.47E-04	U	P38197	1206	156	109	3
YJV7	**Uncharacterized protein YJL217W**	–	–	+1.8	4.62E-03	U	P40893	1380	173	117	3

All the listed proteins showed a statistical difference of spot volume ratio between the control/BTTR-wt and control/BTTR-L55P with an ANOVA p<0.05.

a) Gene code as in the yeast genome database (www.yeastgenome.org).

b) CM – carbohydrate metabolism; AM – amino acid metabolism; NM – nucleotide metabolism; EM – energy metabolism; LM – lipid metabolism; U – unknown; CRH – cell redox homeostasis; T – translation; TP – transport; PFD – protein folding and degradation.

c) Accession code of the uniprot database (www.uniprot.org).

d) Spot number on the master gel (see fig. 1A).

e) Summary of the protein identification results. The protein and the peptide score as given by the GPS Explorer software (Applied Biosystems). The number of peptides with MSMS data is also given.

### Protein Identification and Gene Ontology Analysis

All spots highlighted in Figure 2A were picked and trypsin digested using the Ettan Spot Handling Workstation and the proteins were identified by MALDI-TOF-TOF MS. With this approach, we were able to identify the corresponding proteins in 73 spots, resulting in the identification of 70 unique proteins (Table 1). For the majority of the identified proteins, the molecular mass and isoelectric points determined on the 2D gel are consistent. In some cases, the same protein is identified in different spots across the 2D gel with different molecular mass and isoelectric point suggesting the presence of post-translational modifications and/or protein isoforms. All spots representing the same protein have a very similar regulation (see for example spot 991 and 994 both identified as alcohol dehydrogenase (ADH1) where a similar trend and fold variation was observed). In seven spots, more than one protein was identified (see Table 1). In some cases, MSMS data allowed the identification of a particular protein isoform (example, spot 613 identified as HSP75). In other cases, this was not possible and thus both protein isoforms are shown in table 1 (for example, spot 1542 identified as enolase 1 and/or enolase 2).

A significant change in protein abundance was clearly detected for spot number 1492, absent from the control (Figure 2D). This spot was unequivocally identified as human TTR. This is a noteworthy observation for two main reasons: first, it shows that our experimental system leads to a high TTR expression level; second, the detection of this expected difference validates the approach we chose to detect quantitative differences in protein abundances.

**Figure 3:**
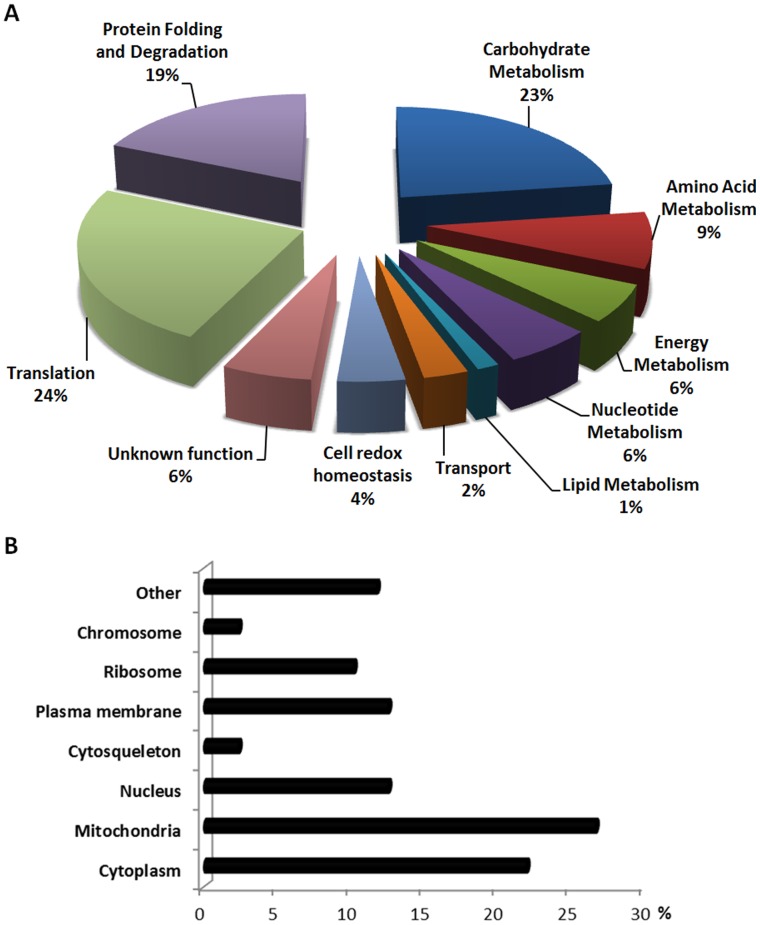
Gene ontology characterization of the identified differentially expressed proteins. (A) Biological function. (B) Cellular location.

The identified proteins were categorised into functional groups and cellular location using Gene Ontology annotations. The 70 identified proteins fell into 10 functional categories (Figure 3A). About 45% are proteins related to cell metabolism, including carbohydrate (16 unique proteins, 23%), amino acid (6 proteins, 9%), energy (4 proteins, 6%), nucleotide (4 proteins, 6%) and lipid metabolism (1 protein, 1%). A significant number of the identified proteins (17 proteins; 24%) are involved in translation, including ribosomal proteins and translational factors. Noteworthy, a high number of the identified differentially expressed proteins are related to protein folding and degradation pathways (13 proteins, 19%). Several of these proteins have been described as stress response ones, involved in the response to an increase protein misfolding (as Hsp70 protein family). Proteins involved in transport (2 proteins, 3%), cell redox-homeostasis (3 proteins, 4%) and proteins with unknown or poorly characterized function (4 proteins, 6%) were also identified.

Concerning cellular location, a high number of identified proteins were from mitochondria (27%), while 22% were from cytoplasm, 12.5% from the plasma membrane and nucleus (Figure 3B).

### Proteome Changes Induced by the Amyloidogenic TTR Variant

**Figure 4:**
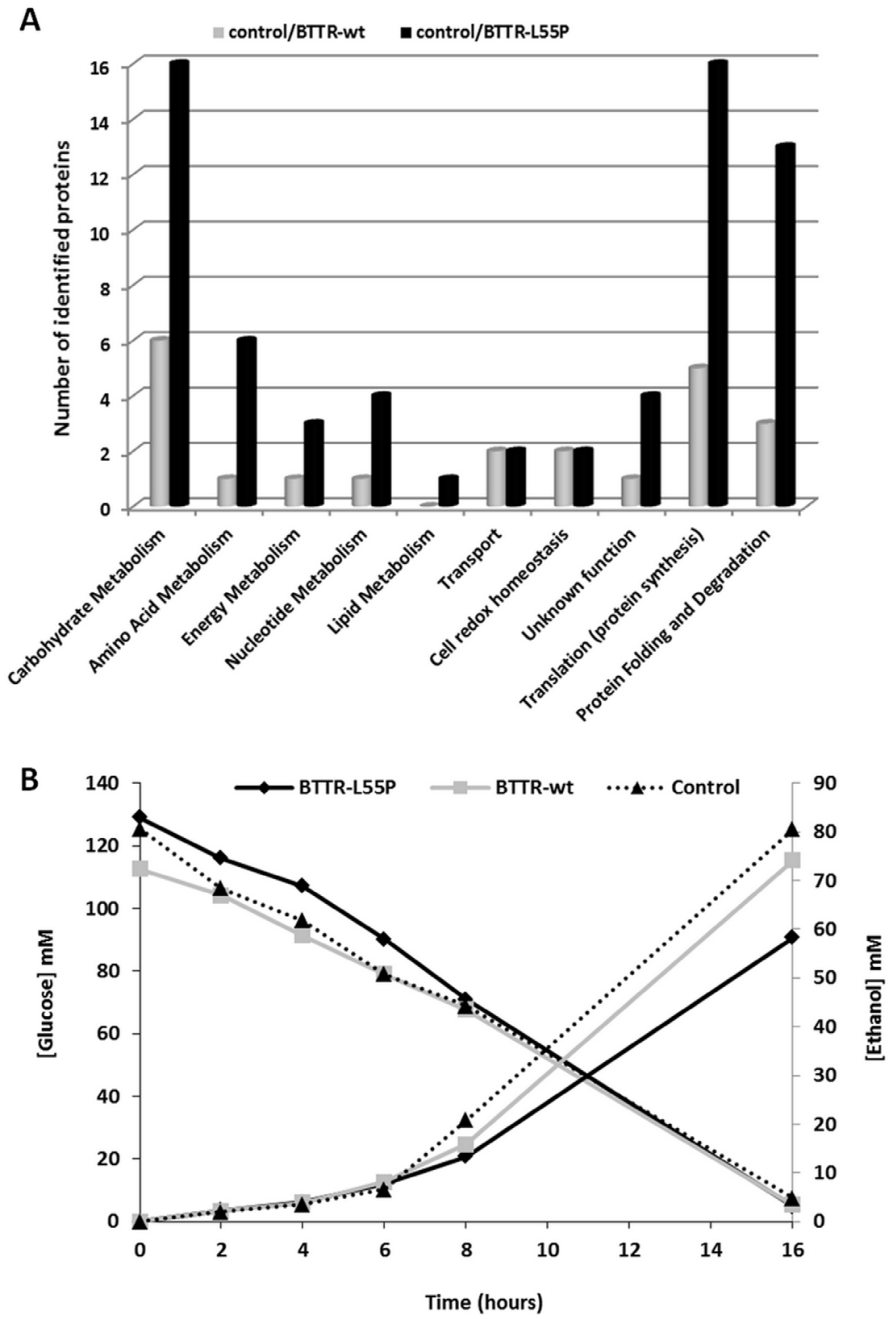
Expression profile in BTTR-wt and BTTR-L55P versus the control. (A) Number of the identified proteins according to the biological function, differentially expressed between the control and BTTR-wt (grey) and between the control and BTTR-L55P (black). Upon the expression of the amyloidogenic TTR variant L55P, a substantial increase in the number of differentially expressed proteins namely in proteins involved in cell metabolism, translation and protein folding and degradation is clearly detected. (B) D-Glucose consumption and ethanol production during cell growth in the control (dotted line), BTTR-wt (grey) and BTTR-L55P (black). A representative analysis is shown.

**Figure 5:**
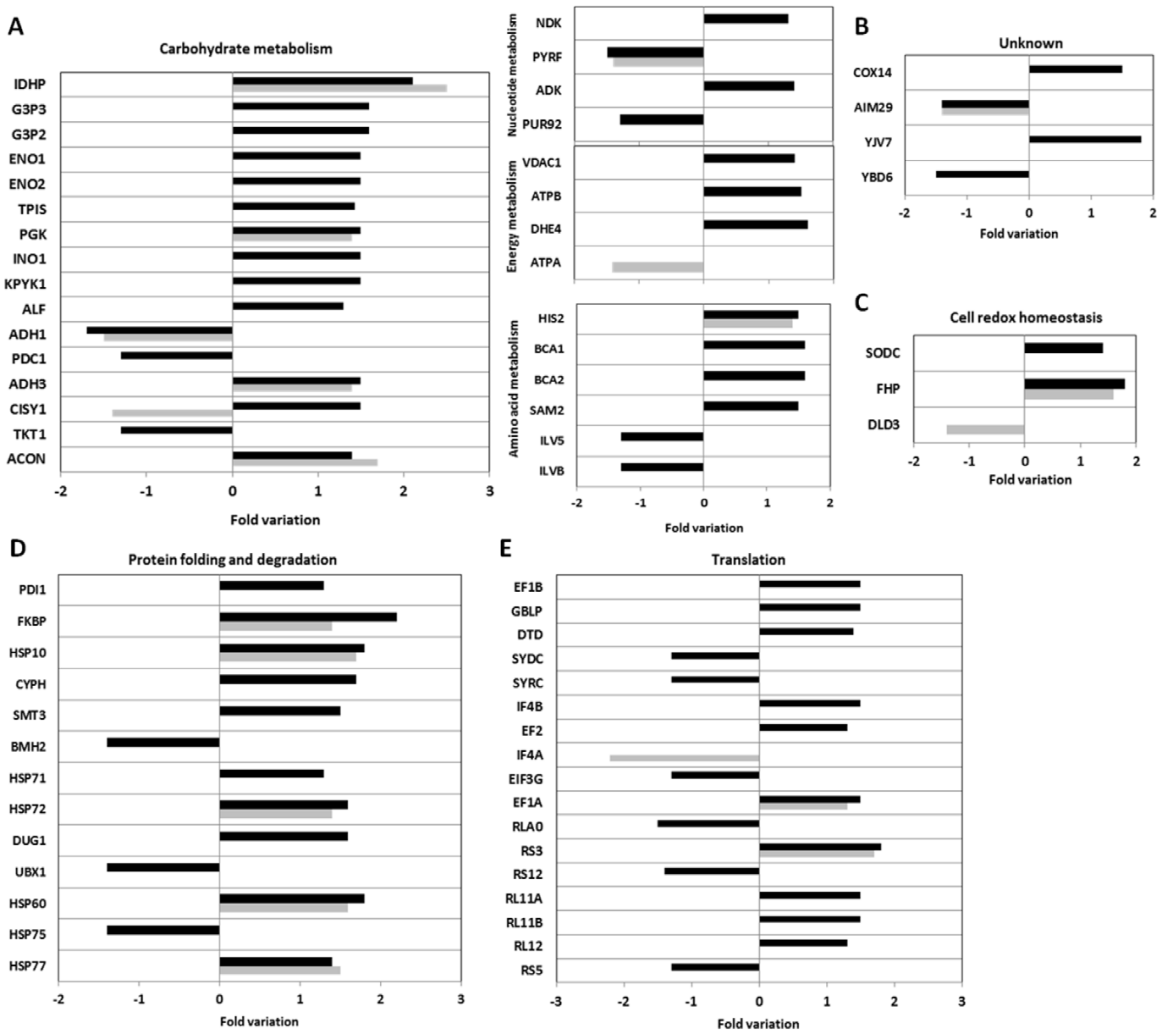
Detailed expression profiles for all the identified differentially expressed proteins, according to its functional categories: (A) cell metabolism; (B) unknown function; (C) Cell redox homeostasis; (D) protein folding and degradation; (E) translation. Grey bars represent fold change in protein expression in BTTR-wt versus the control while black bars represent fold change in protein expression in BTTR-L55P versus the control. The vertical axis indicates the identified protein while the horizontal axis represents the fold variation in protein expression. Additional information for each protein, including full name, can be found in Table 1. For proteins identified in different spots (with slightly different fold variations) the average is represented in the graph.

As described above, a clear differentiation was evident between the proteome of cells expressing the non-amyloidogenic TTR-wt and the highly amyloidogenic TTR-L55P variant. In BTTR-wt, 22 proteins were differentially expressed (Figure 4A), with 15 proteins up-regulated and 7 down-regulated (Figure 5, grey). By contrast, in cells expressing the amyloidogenic TTR-L55P, significant proteome changes were induced with 67 unique proteins differentially expressed, 49 being up-regulated and 18 down-regulated (Figure 4A, detailed in Figure 5, black). To further explore the involvement of the uncovered pathways involved in TTR misfolding, we performed a functional enrichment analysis using DAVID (Database for Annotation, Visualization and Integrated Discovery). This analysis revealed 8 functional clusters with a significantly enrichment score (Table 2). Significant changes in cell metabolism (namely glucose and amino acid metabolism) and also in the regulation of translation and protein synthesis was noticeable. Biological themes related to plasma membrane and mitochondria proteins and a functional enrichment in the molecular chaperones network and in protein refolding was observed. In contrast, the DAVID analysis using the proteins differentially expressed in BTTR-wt revealed three significant clusters only: mitochondrial matrix (FDR of 6.10E-07), tricarboxylic acid cycle (FDR of 3.60E-06) and plasma membrane enriched fraction (FDR of 5.40E-03).

**Table 2:** Functional annotation enrichment analysis of the identified proteins using the Database for Annotation, Visualization and Integrated Discovery (DAVID ) v6.7.

AnnotationCluster	Annotation Terms	FDR
**1**	Glycolysis/Gluconeogenesis	2.70E-07
	Pyruvate metabolic process	2.00E-09
	Alcohol catabolic process	5.50E-09
**2**	Plasma membrane enriched fraction	1.40E-10
**3**	Regulation of translation	1.90E-06
	Cytosolic ribosome	1.10E-03
**4**	Cellular amino acid biosynthetic process	4.10E-05
	Cellular amino acid catabolic process	1.50E-04
	Branched chain family amino acid metabolic process	1.80E-04
	Pantothenate and CoA biosynthesis	3.00E-03
	Glutamate biosynthetic process	5.10E-04
**5**	Molecular chaperone	2.60E-06
	Protein refolding	1.20E-03
**6**	Mitochondrial matrix	6.80E-05
**7**	Metal-binding	1.80E-04
**8**	Nucleotide-binding	5.20E-03

Annotation terms are representative of a particular cluster. FDR – False discovery rate.

**Figure 6:**
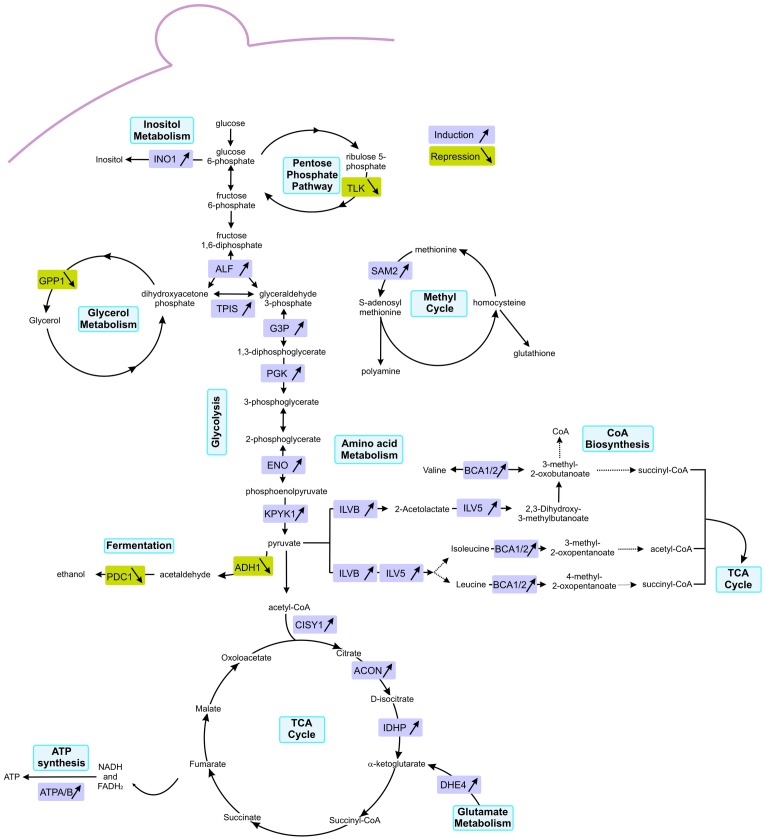
Major metabolic pathways altered in BTTR-L55P. Differentially expressed proteins are highlighted, those induced are framed in purple and those repressed are framed in green. Protein full names can be identified in Table 1.

The high number of the differentially expressed proteins involved in metabolic processes hints that the cell response to protein misfolding stress is accompanied by active metabolic changes. The major metabolic pathways altered are illustrated in Figure 6. Following TTR-L55P expression, we detected an up-regulation of several glycolytic enzymes (Figures 5A and 6), pointing to an increase in glucose catabolism. In addition, the two enzymes that catalyse ethanol formation from pyruvate (PDC1, pyruvate decarboxylase 1 and ADH1, alcohol dehydrogenase) are down-regulated (Figures 5A and 6). This could reflect a shift in the pyruvate fate, from alcoholic fermentation to the TCA cycle and oxidative phosphorylation. Indeed, the TCA cycle enzymes citrate synthase (CISY1) aconitate hydratase (ACON) and isocitrate dehydrogenase (IDHP), which are responsible for the synthesis of α-ketoglutarate from acetyl-CoA, are up-regulated in BTTR-L55P in compassion to control cells. Consistently, an increased abundance of ATP synthase (ATPB) and a down-regulation of the pentose phosphate pathway enzyme transketolase (TKT1) and (DL)-glycerol-3-phosphatase 1 (GPP1), an enzyme involved in glycerol synthesis, was detected. Altogether, these results point to the channelling of glucose catabolism through the TCA cycle, leading to an increase ATP production via cell respiration (Figure 6). Notably, this metabolic change is not apparent in BTTR-wt (Figure 5A in grey). In these cells, D-glucose consumption and ethanol production are similar (Figure 4B).

The increased ATP demand is surely related with the higher energy needed to actively refold or degrade misfolded proteins. In fact, a similar metabolic change was apparent in the response to heat shock that is also characterized by an increased protein unfolding and aggregation [22]. An undesirable by-product of an increased ATP formation via cell respiration is the formation of reactive oxygen species such as superoxide anion which may activate the oxidative stress response. This explains the up-regulation found exclusively in BTTR-L55P of superoxide dismutase [Cu-Zn] (SODC). These data may suggest a link between protein misfolding and oxidative cellular stress derived from a higher ATP demand. It has been show that oxidative modifications may facilitate aggregation of amyloidogenic proteins [23]. Upon aging, cellular defences towards oxidative stress are compromised and oxidative protein modifications are also likely to accumulate, which may be synergistically linked to the disease onset [2].

Another major set of differentially expressed proteins are involved in translation, with a complex pattern of expression (6 proteins down-regulated and 10 up-regulated in BTTR-L55P) (Figure 5E). These changes may reflect an adaptive response to cellular stress. It was reported that elongation factors are up-regulated in response to stress conditions, such as oxidative stress [24]. In addition to its canonical role in translation, unique cellular activities, such as nuclear export, cytoskeleton organization and apoptosis, have been attributed to elongation factor protein family in eukaryotes [25]. Interestingly, a potential role in protein quality control and co-translational degradation has been suggested for these proteins [26]–[28]. Elongation factors interact with the 26S proteasome and this association increases when translation is inhibited [26]. Thus, the identified proteins may be important in the cell response to protein misfolding in a more complex way than a simple activation or inhibition of protein synthesis. Although further studies are needed to clarify this issue, our findings provide a good starting point by revealing potential protein targets.

**Figure 7:**
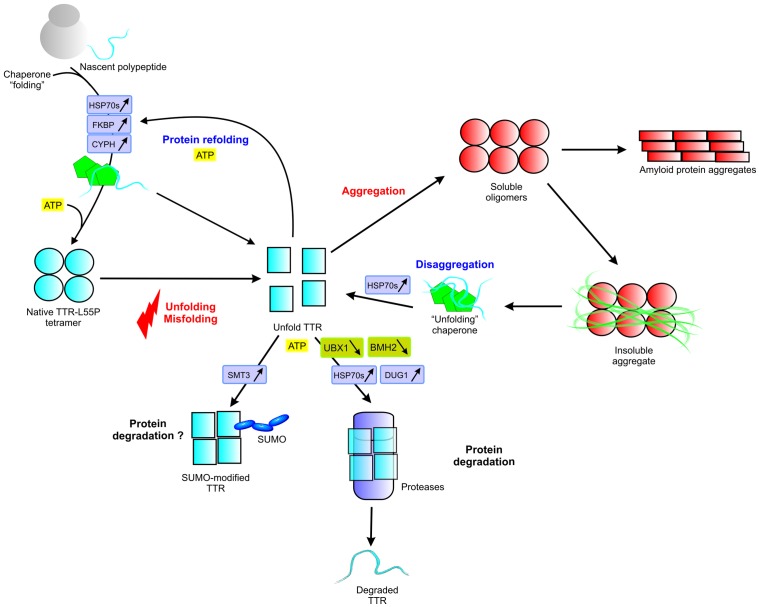
TTR misfolding and protein quality control mechanisms. Differentially expressed proteins are highlighted, those induced framed in purple and those repressed are framed in green. Protein full names are indicated in Table 1.

Several stress-response proteins involved in protein folding and/or degradation were also identified, with 13 proteins differentially expressed in BTTR-L55P (10 up-regulated and only 3 down-regulated, Figure 5D). Some heat shock proteins were also found up-regulated in BTTR-wt by a similar fold variation (HSP77, HSP60, HSP72 and HSP10; Figure 5D, in grey). Interestingly, HSP77, HSP60 and its co-chaperone HSP10 [29] are mitochondrial-resident chaperones, involved in folding of newly imported proteins to the mitochondria. The other identified proteins involved in protein folding and degradation changed their abundance only in BTTR-L55P. The potential role of these proteins in TTR misfolding and aggregation is illustrated in Figure 7.

Two interesting protein revealed by our study are the cyclophilin FK506-binding protein 1 (FKBP) and cyclophilin A (CYPH). These proteins, that have a peptidyl-prolyl-cis-trans isomerase (PPIase) activity, were up-regulated in BTTR-L55P (Figure 5D). FKBP changed its abundance also in BTTR-wt but it increases significantly in BTTR-L55P (1.4 fold vs 2.2, Table 1). The involvement of PPIases in the cell response to protein misfolding and aggregation is still unclear. However, recent experiments implied this protein family in conformational neurodegenerative disorders. It was showed that FKBP52 overexpression reduced the Aβ peptide toxicity and increases the lifespan of flies expressing Aβ peptide, whereas loss of function of FKBP52 exacerbated these Aβ phenotypes [30]. Our previous results revealed that PPIase cyclophilin H is one of the major TTR interactuant in human plasma of ATTR patients [15]. In yeast cells, FKBP was shown to interact with the heat shock factor 1, a major regulator of the cell response to stress conditions such as heat shock and protein misfolding [31]. In addition to FKBP, CYPH was up-regulated by 1.7 fold exclusively in BTTR-L55P. The human homologue of yeast CYPH is a major Aβ-peptide interactuant found in the brain and elevated levels of this protein were reported in human Alzheimeŕs disease brains [32]. Moreover, it was showed that the Aβ oligomeric form has a greater affinity for CYPH, hinting for a relevant role of this protein in protein aggregation [32]. Interestingly, yeast CYPH interacts with several proteasome regulatory subunits and also with ubiquitin and SMT3 (yeast homologue of mammalian SUMO1) [33] suggesting a role in protein degradation associated processes. Altogether, these observations suggest an important role of PPIases in disease development and cellular responses to protein aggregation in the context of conformational disorders and are good protein targets for further studies.

DIGE Screen identified three additional molecular chaperones exclusively in BTTR-L55P: an increased abundance of HSP71 (SSA1 gene) and HSP72 (SSA2 gene) and a reduction in HSP75 (SSB1 gene). Using an animal model of ATTR, it was recently observed that TTR deposition leads to an increase in HSP70 expression [34], in agreement with our 2D-DIGE analysis. However, contrary to our study, the interplay between the different isoforms of the Hsp70 was not revealed, which is highly relevant considering that a functional difference between members of SSA and SSB is apparent [35], [36]. Both classes of chaperones affect *de novo* prion formation in yeast, although with opposite effects [37]. After heat shock, a similar trend was observed with the SSB isoform being down-regulated while the SSA isoforms is up-regulated [35].

Besides HSP70, the endoplasmic reticulum lumen resident protein disulfide isomerase (PDI) was up-regulated exclusively in BTTR-L55P, which appears to be in concordance with an up-regulation of the HSP70 resident endoplasmatic reticulum chaperone BiP in tissues affected with TTR aggregation [38]. Thus, even though a simple model organism was used in this study and TTR was used as a model amyloidogenic protein, it is likely that relevant protein targets, even for familial amyloidosis, was revealed by this screen.

The action of molecular chaperones is an essential first step to avoid protein aggregation. At this point, misfolded proteins are either refolded or degraded, avoiding their accumulation. It is now recognized that some molecular chaperones are also involved, either directly or indirectly, in protein disposal. HSP71, found up-regulated exclusively in BTTR-L55P, and its co-chaperone HSP40 specifically recognize misfolded protein domains and escort them for proteasome degradation [39]. Yeast HSP71 is in fact known to interact with proteasome regulatory subunits (like the 26S RPN 2) [40]. Noteworthy, the ubiquitin-like protein SMT3 was found up-regulated exclusively in BTTR-L55P (Figure 5). This protein displays 50% sequence identity with mammalian SUMO1 protein and is essential for yeast viability. Protein sumoylation is known to be involved in neurodegenerative disorders such as Alzheimer’s [41] and Huntington’s diseases [42] although its exact role is presently controversial. For the Huntington protein, sumoylation renders the protein more soluble and apparently more toxic by inhibiting its aggregation into inclusion bodies [42]. In Alzheimer’s disease, SUMO1 overexpression in tissue cultured cells co-transfected with the APP gene suppresses Aβ fragment accumulation [41]. Studies in human samples of ATTR patients showed that TTR aggregates lead to a significant increase in ubiquitin conjugates and an impairment of the ubiquitin-proteasome system was observed [43].

Two proteins involved in protein folding and degradation were down-regulated exclusively in BTTR-L55P: UBX domain-containing protein 1 (UBX1) and protein BMH2 (Brain Modulosignalin Homologue, member of the ubiquitous 14-3-3 gene family) (Figure 5D, in black). UBX1 is known to interact with proteasome regulatory subunits and with ubiquitylated proteins *in vivo*, being required for the degradation of an ubiquitylated model substrate [44]. Interestingly, UBX1 also interacts with FKBP [45], with several isoforms of the HSP70 family (HSP71 [40] and HSP77 [46]) and also with PDI [47], proteins differentially expressed in response to TTR-L55P expression. Concerning BMH2, it is likely to be related to carbohydrate metabolism and stress response [48], [49]. It was observed that the expression of BMH proteins is altered after exposure to several stress conditions such as heat shock or dithiotreitol [50], [51]. To our knowledge, no association between 14-3-3 proteins and TTR aggregation has been described until now.

Three proteins with unknown or poorly characterized functions were also identified (Figure 5B). In addition to a down-regulation of AIM29 (already detected in BTTR-wt), the UPF0001 protein YBL036C was also down-regulated, while the uncharacterized protein YJL217W and cytochrome c oxidase assembly protein COX14 increase its abundance. The biological function of these proteins is not yet known. Some data seem to relate the YJL217W protein in the regulation of enolase1 [52]. This could be related with the detected increase in glucose catabolism and, considering that yeast enolase1 also function as a heat shock protein [53], with the misfolding of TTR-L55P. This is an interesting hypothesis that requires further investigation, feasible in yeast considering that the ENO1 yeast null mutant is viable. COX14 Is an integral mitochondria membrane protein and a yeast null mutant for this protein displays a respiratory growth deficiency [54]. Thus, the increased expression of this protein may results in an improved mitochondria function. No significant human homology was found for this protein. Finally, UPF0001 protein YBL036C also increased significantly its abundance in BTTR-L55P. A BLAST homology search revealed that this yeast protein shares 43% identity with proline synthase co-transcribed bacterial homolog protein, whose function is nevertheless not yet known.

### Concluding Remarks

The proteome response to protein misfolding and aggregation is a key factor in understanding onset and evolution of conformational disorders. By uncovering specific proteins and pathways effective therapeutic strategies may be devised. In this study, we performed a high-throughput proteomics analysis using the 2D-DIGE technology to screen changes in protein abundance upon the expression of an amyloidogenic protein (TTR-L55P variant) in comparison with the expression of the non-amyloidogenic version of the same protein (TTR-wt). With this approach, around 1800 individual protein spots were quantitatively analyzed. Our results showed a clear cut differentiation between the expression of TTR-wt and TTR-L55P, distinguishing the cell response to the expression of a heterologous protein from the cell response to protein misfolding and aggregation. The proteome changes observed by the expression of TTR-wt and TTR-L55P share about 20 proteins in common that may be attributed to the expression of a heterologous protein in yeast. Expression of the amyloidogenic TTR-L55P caused changes in about 50 exclusive proteins that may be specifically associated to amyloidogenic behavior and protein aggregation. Several heat shock protein (as HSP70 and HSP60) and members of PPIase family (cyclophilin A and FKBP) are up-regulated upon TTR-L55P expression. In addition, several other processes were influenced by the misfolding and aggregation of an amyloidogenic protein such as carbohydrate and amino acid metabolism, energy production, translation and oxidative stress response. The expression of the amyloidogenic TTR variant causes a metabolic shift towards energy production via mitochondrial cell respiration which is related to the high energy demand of refolding and degradation pathways. This in turn may promote oxidative stress. The up-regulation of mitochondrial chaperones (HSP60, HSP77, HSP10, protein disulfide-isomerase) is also consisted with increased oxidative stress and higher energy demands needed to face misfolding and aggregation.

Although in transthyretin amyloidosis TTR accumulates as amyloid deposits in the extracellular space, we believe that TTR is a good amyloidogenic protein model to investigate the cellular responses to the general misfolding and aggregation problem. TTR has a high number of amyloidogenic point mutations and thus the proteome response may be investigated with different amyloidogenic potential against a defined genetic background. It was recently discovered that intracellular signaling mechanisms related to extracellular TTR aggregates in ATTR was elicited [34], [38], [43]. Notably, similarly to our results, changes in the chaperone network (such as HSP70 protein family and endoplasmatic resident chaperones) and degradation processes were detected [38], [43]. Moreover, a striking parallel was found on the specific increased expression of PPIases in yeast expressing TTR-L55P, found to be a specific TTR interacting partner only in symptomatic ATTR subjects [15]. Yeast can thus be used as a model to investigate TTR effects on living cells that is highly relevant in the context of ATTR and other misfolding diseases. Moreover, more than 100 TTR point mutants are known to be associated to amyloid disease and the availability of a complete gene set collection of yeast single gene deletion mutants shows its potential for large scale screening.

## Materials and Methods

### Bacteria, Yeast Strains and Culture Conditions

*Escherichia coli* strain used (DH5α, F-; *rec*A1; *end*A1; *thi*-1; *gyr*A96; hsdR17; *sup*E44; *rel*A1;φ89d; *lac*Z; DM15 λ-) was cultured in LB medium [1% (w/v) NaCl, 1% (w/v) tryptone, 0.5% (w/v) yeast extract] at 37°C. Solid LB medium contained 2% (w/v) agar. Transformed strains, carrying the plasmids, grew in LB medium supplemented with 0.1 mg.ml^−1^ ampicillin. *Saccharomyces cerevisiae* strain used was the BY4741 (genotype BY4741 *MATa*; *his3*Δ1; *leu2Δ0*; *met15Δ0*; *ura3Δ0*) from Euroscarf collection (Frankfurt, Germany). Strains were kept in YPGlu agar slopes [0.5% (w/v) yeast extract, 1% (w/v) peptone and 2% (w/v) D-glucose and 2% (w/v) agar] at 4°C and cultured in liquid YPGlu medium at 30°C. BY4741 strain carrying the TTR expression plasmids were cultured in YNB minimal medium without uracil [0.67% (w/v) yeast nitrogen base, 2% (w/v) D-glucose and 0.025% (w/v) L-methionine, L-histidine, L-leucine].

### Plasmids and Yeast Transformation for TTR Expression

Plasmids p426GPD carrying the different TTR genes (TTR-wt and TTR-L55P) were a king gift of Dr. Tiago Outeiro (Cell and Molecular Neuroscience Unit, IMM, Portugal). Plasmid DNA extraction was performed using the Wizard Plus SV Minipreps DNA Purification System (Promega), following the manufacturer’s instructions. DNA concentration was evaluated spectrophotometrically at 260 nm and purity was assessed by standard procedures. BY4741 yeast strain was transformed by the lithium acetate method, and transformants were selected on minimal agar plates deficient in uracil, following standard procedures [55]. Yeast cells were also transformed with the p426GPD vector without the inserted gene as control. Yeast growth curves were monitored at 640 nm and phenotypic growth assays were carried out by spotting 3 µl of late-exponential-phase culture, sequentially diluted (approx. 2000–20 cells), in selective medium. Growth was recorded after 2 days at 30°C.

D-Glucose and ethanol were enzymatically assayed during cell growth using the D-Glucose assay kit and Ethanol assay kit (NZYTech), following the manufacturer instructions.

### Western Blotting and Protein Aggregate Filtration Assay

TTR expression levels were relatively quantified by western blot using an anti-human TTR polyclonal antibody (SC 13098, Santa Cruz Biotechnology). Protein extraction was performed by glass bead lysis as previously described [56] and protein concentration was determined using the Bio-Rad Bradford assay reagent. Proteins (30 µg per lane) were separated using denaturant urea gel electrophoresis in a Mini-protean 3 system (Bio-Rad). Proteins were transferred to PVDF membranes (Immobilon-P PVDF, Millipore), using the Mini Trans-Blot system (Bio-Rad) and the membrane was blocked overnight at 4°C in TBS-T (50 mM Tris and 150 mM NaCl, pH 7.5 with 0.1% (v/v) Tween 20) containing 5% (w/v) skimmed milk. TTR polyclonal antibody was used at a dilution of 1∶5000. Ponceau S staining was used to monitor protein transfer and to confirm that equal amount of protein were loaded in each lane. Washes, secondary antibody and detection procedures were performed using the BM Chemiluminescence Western Blotting Kit (Roche), following the manufacturer’s instructions.

Protein aggregate filtration assay was performed essentially as described [57], [58], with slight modifications. Briefly, yeast cells expressing TTR variants were suspended in modified lysis buffer (50 mM Tris containing 5 mM MgCl_2_, 0.1 mM EDTA, 2 mM PMSF and proteases cocktail inhibitor) and lysed with glass beads as described [56]. Cell ghosts were removed by centrifugation at 2500 g for 5 min at 4°C. Supernatants were collected as the crude protein extract and protein concentrations in all samples were equalized. 150 µl Of the crude extract was centrifuged at 14000 g for 30 min at 4°C to separate the soluble from aggregated proteins. The pellet fractions, containing the insoluble aggregated proteins, were suspended in 2% (w/v) SDS or in lysis buffer. Samples were filtered on a dot-blot filtration unit (Bio-Rad) through a nitrocellulose membrane (0.2 µm pore size) that was previously blocked and pre-equilibrated with 2% (w/v) SDS. Filters were washed twice with 0.1% (w/v) SDS (except the samples suspended in lysis buffer, which were washed with the same buffer). TTR aggregates were imunodetected using the anti-human TTR polyclonal antibody at a dilution of 1∶5000. To assess the assay specify toward TTR protein aggregates and not soluble TTR, the crude extract and the soluble protein fraction were analysed through a nitrocellulose membrane (as a regular dot-blot assay) and a blocked nitrocellulose membrane.

### Protein Sample Preparation and CyDye Protein Labeling

For 2D-DIGE analysis, cells were collected at mid-log phase of growth, harvested by centrifugation and suspended in 2D-DIGE labeling buffer [7 M urea, 2 M thiourea, 4% (w/v) CHAPS and 30 mM Tris] containing proteases inhibitors (PMSF and proteases cocktail inhibitor, Sigma). Yeast cells were lysed using glass beads as previously described [56]. All samples (four biological replicates) were processed in parallel. Protein extracts were clarified by centrifugation at 12000 g for 15 min at 4°C. The cell lysate pH was then carefully adjusted to 8.5 with NaOH and afterwards protein concentration was determined using the 2D Quant Kit (GE Healthcare) with BSA as standard. Prior to electrophoresis, protein extracts were labeled with CyDyes™ (GE Healthcare), following the manufacturer instructions. Briefly, proteins were labeled by mixing 240 pmol of fluorochromes (Cy3 or Cy5) with 30 µg of protein and incubated on ice for 30 min in the dark. Lysine (1 µl, 10 mM) was then added to quench the reaction and the samples were left on ice for 10 min in the dark. A pooled internal standard was performed by mixing 15 µg of each sample analyzed that was labeled with Cy2 dye and included in all gel runs. A dye swap was used between Cy3 and Cy5 to avoid problems associated with preferential labeling.

### 2D Gel Electrophoresis

For 2D gel electrophoresis, the two samples to be run on the same gel plus the internal standard were mixed before adding 2x lysis buffer [7 M urea, 2 M thiourea, 4% (w/v) CHAPS, 6 µl.ml^−1^ DeStreak reagent (GE Healthcare)] and 2% (v/v) ampholytes immobilized pH gradient buffer (pH 3–10 NL, GE Healthcare) to a final volume of 125 µl. Isoelectric focusing was carried out on pH 3–10 IPG-strips (24 cm, non-linear gradient; GE Healthcare) using the IPGphor3 system from GE Healthcare. ImmobilineDryStrips were rehydrated overnight with 450 µl DeStreak Rehydration Solution (GE Healthcare), complemented with 1.5% ampholytes, before cup-loading of proteins and IEF on an EttanIPGphor Manifold (GE Healthcare). The migration was performed at 20°C (60 V for 2 h; gradient from 60 to 500 V for 5 h; hold 500 for 1 h, gradient from 500 to 1000 for 3 h; hold 1000 V for 1 h; gradient from 1000 V to 8000 V for 4 h, hold 8000 V until 64 000 Vh). After the IEF, IPGstrips were equilibrated twice for 15 min in equilibration buffer [50 mM Tris-HCl pH 8.8, 6 M urea, 30% (v/v) glycerol, 2% (w/v) SDS and 0.002% (w/v) bromophenol blue] supplemented with 1% (w/v) DTT and then with 2.5% (w/v) iodoacetamide. Second-dimension SDS-PAGE was performed using 1.0 mm large-format 12.5% polyacrilamide resolving gel and run at 20°C overnight with 1.5W per gel, using the EttanDALTtwelve system (GE Healthcare). Glass plates used for picking gels were treated with Bind-Silane solution [80% (v/v) ethanol, 2% (v/v) acetic acid and 0.1% (v/v) Bind-Silane (GE Healthcare)] before casting. The gels ran simultaneously, with a dye switching between repetitions, plus the internal standard. In the end, ninety micrograms of proteins were loaded on each 2D gel.

### Scanning and Image Analysis

2D-DIGE gels were scanned at a pixel size of 100 µm using a Typhoon Imager 9400 (GE Healthcare) at three different wavelengths corresponding to the different CyDyes. Gel images were exported into Progenesis SameSpot V3 image analysis system (Nonlinear Dynamics), where quantitative analysis of protein spots was performed. Following automatic and subsequent manual editing, aligning and matching procedures as part of the Progenesis SameSpot workflow, ANOVA p-values between the samples were calculated within the Progenesis SameSpot software. Variation of protein expression was considered statistically significant if the absolute abundance variation was at least 1.3-fold between spots of any experimental group with a p<0.05 by ANOVA. Unsupervised PCA correlation analysis was performed using the statistical tool within the gel analysis software. Clustering of each sample was based on the expression pattern of each spot with a significant ANOVA p-value. The spots of interest were visually checked and selected for protein identification by mass spectrometry.

### Spot Handling and Mass Spectrometry Analysis

Spots of interest were excised from gels using the EttanSpot Picker from the Ettan Spot Handling Workstation (GE Healthcare). After washing and desalting in 50 mM ammonium bicarbonate, 50% (v/v) methanol and 75% (v/v) acetonitrile, spots were then digested with Trypsin Gold for 6 h at 37°C (MS grade, Promega, 5 µg.mL^–1^ in 20 mM ammonium bicarbonate) using the Ettan Digester robot from the same workstation. Supernatants were collected, vacuum dried and peptides were thoroughly dissolved in 3 µl of 50% (v/v) acetonitrile containing 0.1% (v/v) trifluroacetic acid. Peptides were spotted on MALDI targets using a matrix consisting of 7 mg.ml^−1^ of α-cyano-hydroxycynamic in 50% (v/v) acetonitrile containing 0.1% (v/v) trifluroacetic acid. Monoisotopic peptide mass determinations were carried out using the MALDI-TOF/TOF 4800 Plus mass spectrometer (Applied Biosystems). For spots with a low signal, the peptide mixture was purified and concentrated using home-made chromatographic microcolumns using GELoader tips packed with POROS R2 as described [59]. MS experiments were performed in positive reflectron mode for monoisotopic peptide mass determination. The mass spectrometer was externally calibrated using the 4700 Calibration Mix (Applied Biosystems). MS spectra were collected in a result-independent acquisition mode, typically using 1000 laser shots per spectra and a fixed laser intensity of 3100 V. For tandem experiments, fifteen of the strongest precursors were selected for MS/MS, the weakest precursors being fragmented first. MS/MS analyses were performed using CID (Collision Induced Dissociation) with 1 kV collision energy at 1 × 10^6^ torr air pressure. Spectra were collected using a fixed laser intensity of 4200 V and 2000 laser shots. Raw data were generated by the 4000 Series Explorer Software v3.0 RC1 (Applied Biosystems) and tryptic peptide contaminant *m/z* peaks resulting from trypsin auto-digestion were excluded when generating the peptide mass list used for comparison with the theoretical tryptic digest. Proteins were identified by the GPS explorer (Applied Biosystem) using the following search parameters: 1) carboxyiodomethylation of cysteine residues and methionine oxidation were taken as fixed and variable modifications, respectively; 2) tolerance of one missed cleavage; 3) maximum error tolerance of 50 ppm for MS data and 0.3 Da for the MS/MS data. Protein identifications were further confirmed using the ProteinPilot software (Applied Biosystem).

#### Gene ontology and pathway analysis

The identified proteins were categorized into functional groups using the gene ontologies annotations available at the Universal Protein Resource Protein Knowledge database (UniProtKB; http://www.uniprot.org/), Kyoto Encyclopedia of Genes and Genomes (KEGG; http://www.genome.jp/kegg/) and Saccharomyces Genome Database (www.yeastgenome.org/). BLAST searches were performed using the UniProtKB tools. A functional annotation enrichment analysis was performed using DAVID (Database for Annotation, Visualization and Integrated Discovery) [60], [61], available at http://david.abcc.ncifcrf.gov/. In addition to gene ontology, the PIR (Protein Information resource), COG (Clusters of Orthologous Groups), Uniprot, KEEG, Interpro and SMART (Single Modular Architecture Research Tool) databases were used within DAVID to generate biological theme by grouping like terms, thereby creating functional annotation clusters. The results were manually checked for significant enriched terms.
